# Leak current, even with gigaohm seals, can cause misinterpretation of stem cell-derived cardiomyocyte action potential recordings

**DOI:** 10.1093/europace/euad243

**Published:** 2023-08-08

**Authors:** Alexander P Clark, Michael Clerx, Siyu Wei, Chon Lok Lei, Teun P de Boer, Gary R Mirams, David J Christini, Trine Krogh-Madsen

**Affiliations:** Department of Biomedical Engineering, Cornell University, Ithaca, NY, USA; Centre for Mathematical Medicine and Biology, School of Mathematical Sciences, University of Nottingham, Nottingham, UK; Department of Physiology and Pharmacology, SUNY Downstate Health Sciences University, Brooklyn, NY, USA; Institute of Translational Medicine, Faculty of Health Sciences, University of Macau, Macau, China; Department of Biomedical Sciences, Faculty of Health Sciences, University of Macau, Macau, China; Department of Medical Physiology, Division of Heart and Lungs, University Medical Center Utrecht, Utrecht, The Netherlands; Centre for Mathematical Medicine and Biology, School of Mathematical Sciences, University of Nottingham, Nottingham, UK; Department of Biomedical Engineering, Cornell University, Ithaca, NY, USA; Department of Physiology and Pharmacology, SUNY Downstate Health Sciences University, Brooklyn, NY, USA; Department of Physiology and Biophysics, Weill Cornell Medicine, 1300 York Avenue, Box 75, Room C501D, New York, 10065 NY, USA; Institute for Computational Biomedicine, Weill Cornell Medicine, 1300 York Avenue, Box 75, Room C501D, New York, 10065 NY, USA

**Keywords:** Induced pluripotent stem cells, Patch clamp, Arrhythmias, Ion channels, Computer simulation

## Abstract

**Aims:**

Human-induced pluripotent stem cell-derived cardiomyocytes (iPSC-CMs) have become an essential tool to study arrhythmia mechanisms. Much of the foundational work on these cells, as well as the computational models built from the resultant data, has overlooked the contribution of seal–leak current on the immature and heterogeneous phenotype that has come to define these cells. The aim of this study is to understand the effect of seal–leak current on recordings of action potential (AP) morphology.

**Methods and results:**

Action potentials were recorded in human iPSC-CMs using patch clamp and simulated using previously published mathematical models. Our *in silico* and *in vitro* studies demonstrate how seal–leak current depolarizes APs, substantially affecting their morphology, even with seal resistances (*R*_seal_) above 1 GΩ. We show that compensation of this leak current is difficult due to challenges with obtaining accurate measures of *R*_seal_ during an experiment. Using simulation, we show that *R*_seal_ measures (i) change during an experiment, invalidating the use of pre-rupture values, and (ii) are polluted by the presence of transmembrane currents at every voltage. Finally, we posit that the background sodium current in baseline iPSC-CM models imitates the effects of seal–leak current and is increased to a level that masks the effects of seal–leak current on iPSC-CMs.

**Conclusion:**

Based on these findings, we make recommendations to improve iPSC-CM AP data acquisition, interpretation, and model-building. Taking these recommendations into account will improve our understanding of iPSC-CM physiology and the descriptive ability of models built from such data.

## Graphical Abstract

**Graphical abstract:**
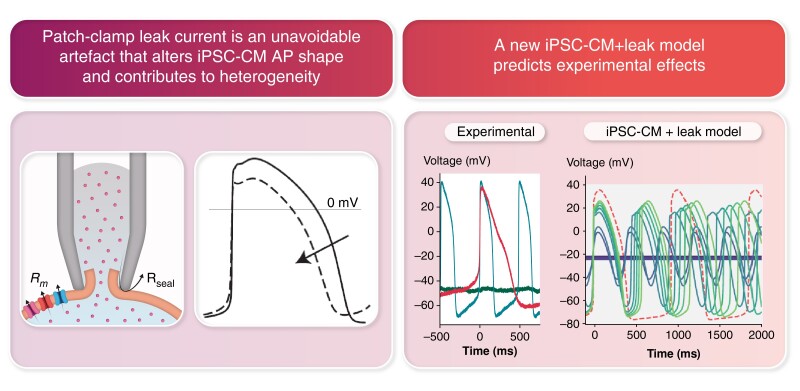

**What’s new?**

- Human-induced pluripotent stem cell-derived cardiomyocytes (iPSC-CMs) are an emerging tool in the study of cardiac arrhythmia mechanisms.
- Their immature and heterogeneous action potential phenotype complicates the interpretation of experimental data and has slowed their acceptance in industry and academia.
- We suggest that the leak current caused by imperfect pipette membrane seal during single-cell patch clamp experiments is partly responsible for causing this heterogeneity and the appearance of immaturity.
- Using *in vitro* experiments and computational modelling, we show that this seal–leak current affects iPSC-CM action potential morphology, even under ‘ideal’ experimental conditions.
- Based on these findings, we make recommendations that should be considered when interpreting, analysing and fitting iPSC-CM data.

## Introduction

Human-induced pluripotent stem cell-derived cardiomyocytes (iPSC-CMs) are a renewable and cost-effective model for studying genetic disease mechanisms,^1,2^ drug cardiotoxicity,^3^ and inter-patient variability.^4^ Computational approaches have been developed to translate experimental results from iPSC-CMs to make predictions in adult cardiomyocytes.^5^ Such work attempts to bridge the critical gap that remains between the physiology of iPSC-CMs and excised adult human cardiac cells.

Whilst iPSC-CMs have transformed many areas of cardiac arrhythmia research, phenotypic heterogeneity and immaturity continue to stymie their potential impact.^6,7^ Investigating sources of these limitations and their biological implications is important as iPSC-CMs (and mechanistic models describing their behaviour) are used to inform increasingly complex clinical decisions.^8,9^ Studies of iPSC-CMs in a single-cell patch clamp context have indicated that their depolarized, highly varying resting membrane potential is primarily due to decreased inward rectifier potassium current (*I_K_*_1_) and increased funny current (*I_f_*) compared with adult cardiomyocytes.^10^

Recently, findings from Horváth *et al*.^11^ and Van de Sande *et al*.^12^ indicate that the heterogeneous and depolarized resting membrane potential is also due, far more than previously thought, to a simple seal–leak current (*I*_leak_). Relative to electrically coupled iPSC-CMs, they show a substantial depolarization in the resting membrane potential in isolated iPSC-CMs despite some cells having similar *I_K_*_1_ densities to human adult cardiomyocytes.^11^ These findings indicate that *I*_leak_ plays an important role in iPSC-CM AP morphology during single-cell patch clamp experiments.

*I*_leak_ is inversely proportional to the seal resistance (*R*_seal_) formed between the micropipette tip and cell membrane during patch clamp experiments. A sufficiently large *R*_seal_ is expected to limit *I*_leak_’s effect on AP morphology. Upon reviewing single-cell electrophysiological iPSC-CM studies, including those used to build iPSC-CM computational models,^13–15^ we found that studies do not report either an *R*_seal_,^10,16–19^ a >1 GΩ *R*_seal_ acceptance criteria,^20^ or an average *R*_seal_ < 3 GΩ.^11,12^

In this study, through *in vitro* experiments and computational modelling, we show that *I*_leak_ affects iPSC-CM AP morphology, even above the *R*_seal_ values usually deemed acceptable in the literature. We show that *R*_seal_ cannot be easily compensated because it cannot be accurately measured during an experiment. Additionally, we posit that the background sodium current (*I*_bNa_) in iPSC-CM models may be overestimated and mimic the effects of leak on AP morphology. Ultimately, we argue that leak current should be considered when interpreting, analysing, and fitting iPSC-CM AP data.

## Methods

### Modelling *I*_leak_

We added a leak equation to the Kernik^13^ and Paci^14^ iPSC-CM and ToR-ORd^21^ adult cardiomyocyte models. Knowing that leak acts as a depolarizing current in iPSC-CM studies and lacking information about specific charge carriers, we modelled *I*_leak_ as having a reversal potential of zero:^22,23^


(1) $$I_{\mathrm{leak}}=\frac{1}{R_{\mathrm{seal}}}V=g_{\mathrm{seal}}V,$$

where *R*_seal_ is the seal resistance and *V* denotes the membrane potential. The inverse of *R*_seal_ is the conductance, *g*_seal_. Note that more complicated equations for leak current (non-linear, and/or with a non-zero reversal potential) may be required in experiments where CaF_2_ seal enhancer is used.^24^

The effect of *I*_leak_ on the evolution of *V* was modelled as follows:


(2) $$\frac{dV}{dt}=-\frac{1}{C_{m}}(I_{\mathrm{ion}}+I_{\mathrm{leak}}),$$

where *I*_ion_ represents the sum of transmembrane currents and *C_m_* is the membrane capacitance. *C_m_* was set to 50 pF (the experimental average from the cells used in the present study) for the Kernik and Paci simulations, and for ToR-ORd, a value of 50 or 153 pF (the ToR-Ord baseline capacitance) was used unless specified otherwise.

### Electrophysiological setup and data analysis

Perforated patch clamp experiments were conducted following a previously described protocol (see *Supplementary Methods* for more details).^25^

After contact was made with a cell and a seal of >300 MΩ was formed, the perforating agent slowly decreased the access resistance to the cell (usually 10–15 min). This low *R*_seal_ acceptance criterion was selected because we wanted to explore seal–leak effects above and below 1 GΩ. A series resistance (*R_s_*) of 9–50 MΩ was maintained for all experiments. In this study, we used all cells from Clark *et al*.^25^ with membrane resistance (*R_m_*) and *R_s_* measurements acquired before and after current clamp recordings and that did not produce spontaneous alternans (*n* = 37 out of 40 cells). *R_m_*, *C_m_*, and *R_s_* values were measured at 0 mV within 1 min prior to the acquisition of current clamp data.

All action potential (AP) features were calculated using a 10-s sample of current clamp data. The minimum potential (MP) was taken as the minimum voltage during this 10-s span. Maximum upstroke velocity (*dV*/*dt*_max_), AP duration at 90% repolarization (*APD*_90_), and cycle length (CL) were averaged over all APs in the 10 s sample.

### *R*_in_ as an estimate of *R*_seal_

We calculate *R*_seal_ using a small test pulse in voltage clamp mode:^26^


(3) $$R_{\mathrm{seal}}=\frac{\Delta V_{\mathrm{cmd}}}{\Delta I_{\mathrm{out}}}.$$

**Figure 1:**
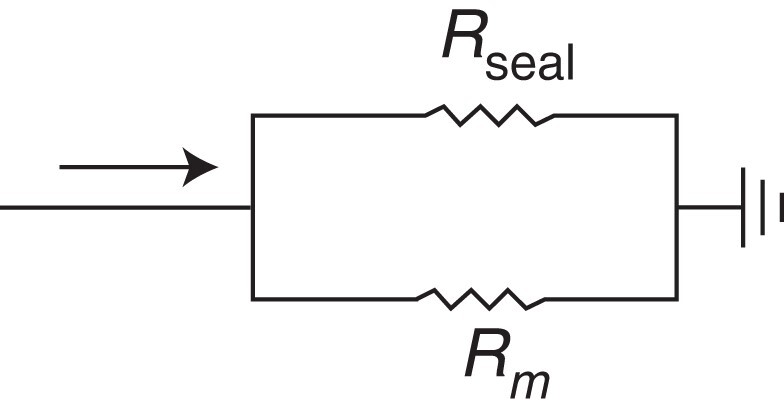
*R*_seal_ cannot be measured directly once access is gained. Once access is gained, we can only measure the combined resistance *R*_in_, which is equal to the parallel resistances of *R*_seal_ and *R_m_* [Eq. (4)]. The presence of *R_m_* introduces uncertainty when *R*_in_ is used to approximate *R*_seal_, making it difficult to accurately correct for leak current effects. For simplicity, we have omitted other elements of this patch clamp diagram (e.g. series resistance and capacitance).

Here, Δ*V*_cmd_ is the applied voltage step, and Δ*I*_out_ is the difference in recorded current from before to during the step. Once access is gained to a cell, it can be difficult to estimate *R*_seal_, as the measured input resistance (*R*_in_) depends on both *R_m_* and *R*_seal_ [Eq. (4); *Figure 1*]. The effect of patch clamp series resistance on *R*_in_ measures was excluded from Eq. (4).


(4) $$1/R_{\mathrm{in}}=1/R_{m}+1/R_{\mathrm{seal}}$$

The smallest *R*_seal_ considered was 300 MΩ, whilst *R_s_* values ranged from 9 to 50 MΩ. An increase of *R*_s_ from 9 to 50 MΩ (a worst-case scenario we never observed) for a cell with a 300 MΩ *R*_seal_ would change *R*_in_ by 13%. So, whilst *R_s_* can change in these experiments, it is unlikely to affect *R*_in_ by more than a few per cent, and *R*_seal_ is likely the predominant parameter affecting changes of *R*_in_.

### Additional methods

Additional methods can be found in the *Supplementary material*.

## Results

### Leak affects human-induced pluripotent stem cell-derived cardiomyocytes action potential morphology even at seal resistances above 1 GΩ

**Figure 2:**
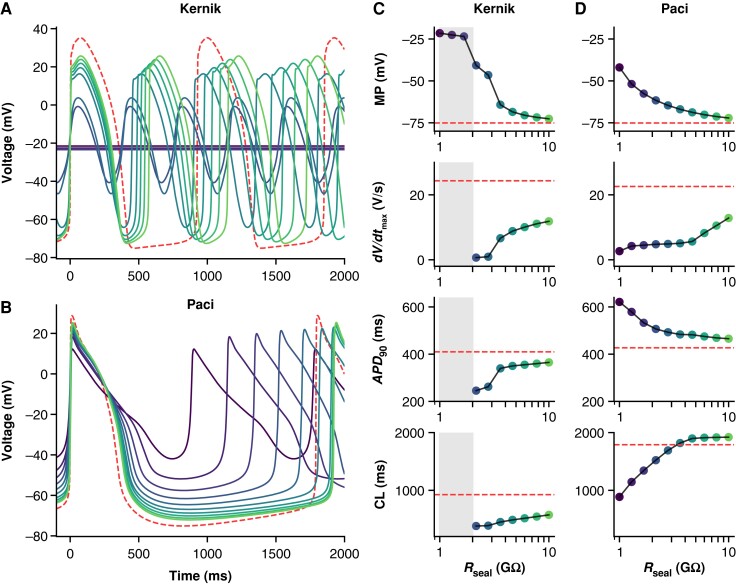
Effect of *R*_seal_ on Kernik and Paci APs. Simulations from the Kernik + leak (*A*) and Paci + leak (*B*) models, each with capacitance set to 50 pF (the experimental average), and *R*_seal_ set to values from 1 to 10 GΩ. The dashed (red) trace shows a baseline (leak-free) simulation. Four AP morphology metrics for the Kernik (*C*) and Paci (*D*) models are plotted against *R*_seal_ (displayed on log-scaled *x*-axis): MP, *dV*/*dt*_max_, *APD*_90_, and CL. Grey boxes denote the *R*_seal_ values where the Kernik model is non-spontaneous. Abbreviations: *APD*_90_, action potential duration at 90% repolarization; CL, cycle length; *dV*/*dt*_max_, maximum upstroke velocity.

To investigate the effects of leak current on AP morphology, we simulated the addition of *I*_leak_ in the Kernik^13^ and Paci^14^ iPSC-CM models (*Figure 2*). Simulated AP recordings show that *I*_leak_ substantially alters AP morphology, even when *R*_seal_ ≥ 1 GΩ, a common threshold used in cardiac patch clamp experiments.^20^ For both models, decreases in *R*_seal_ depolarize the MP and cause a decrease in the *dV*/*dt*_max_, likely due to an incomplete recovery of sodium channels at these depolarized MPs. Indeed, the Kernik model shows a transition to a small amplitude oscillation with very low upstroke velocity when *R*_seal_ < 3 GΩ and then depolarized quiescence when *R*_seal_ < 2 GΩ. *I*_leak_ effects on the *APD*_90_ differ for the two models—decreases to *R*_seal_ cause AP prolongation in the Paci model and AP shortening in the Kernik model. There are also differences in the effect of *R*_seal_ on CL: in the Kernik model, decreases in *R*_seal_ lead to a gradual decrease in CL, whilst in the Paci model, decreasing *R*_seal_ initially has limited effect on CL but then causes shortening as *R*_seal_ decreases below 5 GΩ.

### Leak effects on adult cardiomyocyte action potentials are moderated by different current densities and increased ionic currents

**Figure 3:**
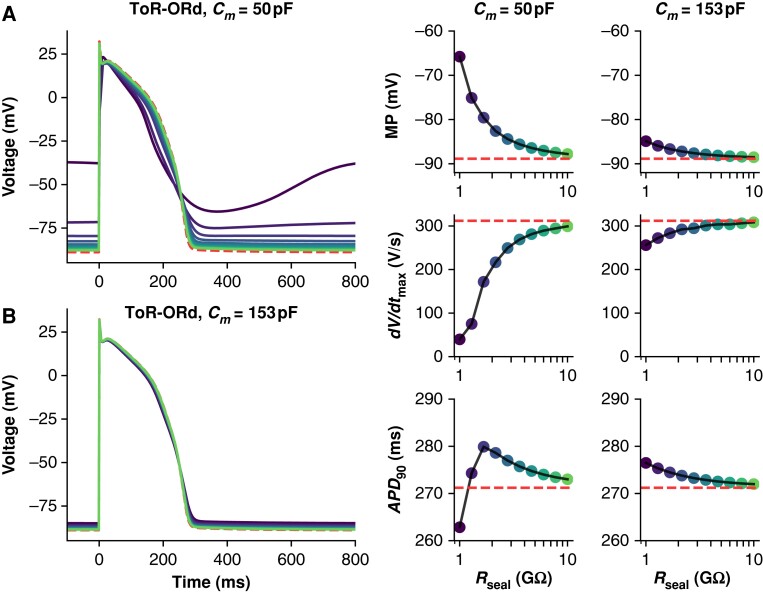
Effect of *R*_seal_ on ToR-ORd adult cardiomyocyte APs at 50 and 153 pF. Simulations from the ToR-ORd + leak model paced at 1 Hz with *C_m_* set to 50 (*A*) and 153 pF (*B*), and *R*_seal_ set to values from 1 to 10 GΩ. The dashed (red) trace shows a baseline (leak-free) simulation. Three AP morphology metrics for the 50 and 153 pF models are plotted against *R*_seal_ (displayed on log-scaled *x*-axis): *APD*_90_, action potential duration at 90% repolarization; *dV*/*dt*_max_, maximum upstroke velocity; MP, minimum potential.

The ToR-ORd adult cardiomyocyte model is also susceptible to *I*_leak_ effects, but the extent depends on cell capacitance (*Figure 3*). Simulations with *C_m_* set to the average iPSC-CM capacitance (50 pF) result in substantial AP morphological changes when *R*_seal_ is between 1 and 2 GΩ. However, when *C_m_* is set to a value in the range of adult human ventricular cardiomyocytes (153 pF), *I*_leak_ has little effect on AP morphology when *R*_seal_ is ≥1 GΩ (*Figure 3B*).

### *R*_seal_ is not stable

Unlike voltage clamp recordings, the effects of *I*_leak_ on AP morphology (measured in current clamp mode) cannot be corrected in post-processing. Current clamp leak compensation is a potential solution to the issue^22,23^ but requires an accurate measure of *R*_seal_ throughout the experiment.

*R*_seal_ cannot be accurately determined after access is gained because measures are contaminated by *R_m_*; such resistance measures are a composite of these two resistances that we nominally refer to as *R*_in_ (see *Figure 1* and Methods). It is, therefore, tempting to measure the value before gaining access and assume it remains unchanged for the duration of an experiment. To investigate this, we considered *in vitro R*_in_ measures taken two times during iPSC-CM experiments. *R*_in_ was measured with 5 mV steps from a holding potential of 0 mV (i.e. the leak reversal potential) before and after acquiring current clamp data. The data are skewed, with a mean of *R*_in_ = 2.71 GΩ and median of *R*_in_ = 0.82 GΩ.

**Figure 4:**
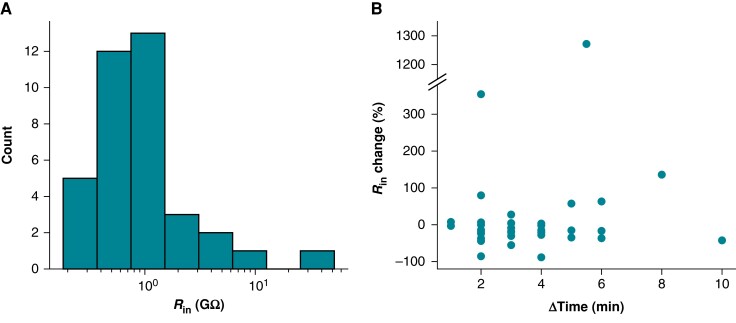
*R*_in_ changes during iPSC-CM experiments. (*A*) Distribution of initial *R*_in_ measurements from iPSC-CMs acquired with a +5 mV step from 0 mV. (*B*) The percentage change in *R*_in_ plotted against the time elapsed between *R*_in_ measurements. The interval between measurements ranged from 1 to 10 min. Time was recorded to the nearest minute, leading to the appearance of banding in the ΔTime measure.

The relative change in *R*_in_ from the first to the second time point was calculated and is plotted against the time elapsed between *R*_in_ measurements in *Figure 4B*. The median change of *R*_in_ is −15%. Because positive and negative changes cancel each other out in these statistics, we also inspected the absolute change, where we found a median of 20%. These data illustrate that *R*_in_ measurements often change over time. If we assume *R_m_* is stable during experiments, this change in *R*_in_ should be attributed to *R*_seal_ and suggests that the average cell’s *R*_seal_ decreases (and therefore *I*_leak_ increases) over time.

### *R*_in_ is not a good approximation of *R*_seal_ at any holding potential

A holding potential of −80 mV is a common choice for approximating *R*_seal_ with *R*_in_ measures. At this potential, sodium, calcium, and several potassium currents are expected to be largely inactive, but contributions from both *I_K_*_1_ and *I_f_* must still be considered. Whilst *I_K_*_1_ is perhaps close to its reversal potential (and therefore small), *I_f_* is not and can play a large role at this voltage.

**Figure 5:**
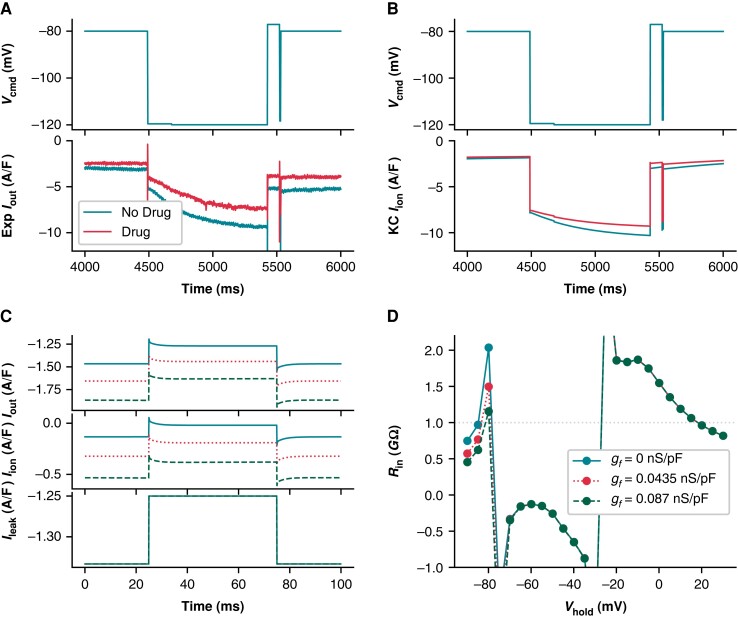
Ignoring the presence of *I_f_* makes it impossible to accurately measure *R*_seal_ after gaining access. (*A*) Voltage clamp data acquired from an iPSC-CM before and after treatment with quinine, which is expected to block 32% of *I_f_* at the concentration used. (*B*) Kernik model response at baseline and with 32% block of *I_f_*. (***C***) Kernik + leak voltage clamp simulations conducted with *R*_seal_ = 1 GΩ, *g_K_*_1_ reduced by 90%, and *g_f_* set to 0 (solid line), 0.0435 (dotted line), or 0.087 nS/pF (dashed line). A voltage step from −80 to −75 mV was applied, as is commonly used to estimate *R*_in_. This *R*_in_ value is sometimes used to approximate *R*_seal_ when the holding potential is near −80 mV. The amplifier-measured (*I*_out_), total transmembrane (*I*_ion_), and leak currents (*I*_leak_) are displayed. The *R*_in_ values calculated based on Δ*I*_out_ are 2.03, 1.50, and 1.16 GΩ for the 0, 0.0435, and 0.087 nS/pF simulations, respectively. (*D*) *R*_in_ values are plotted against holding potential for Kernik + leak models with *R*_seal_ = 1 GΩ and *g_f_* equal to 0, 0.0435, or 0.087 nS/pF. The horizontal dotted line shows the true simulated *R*_seal_ value of 1 GΩ.

**Figure 6:**
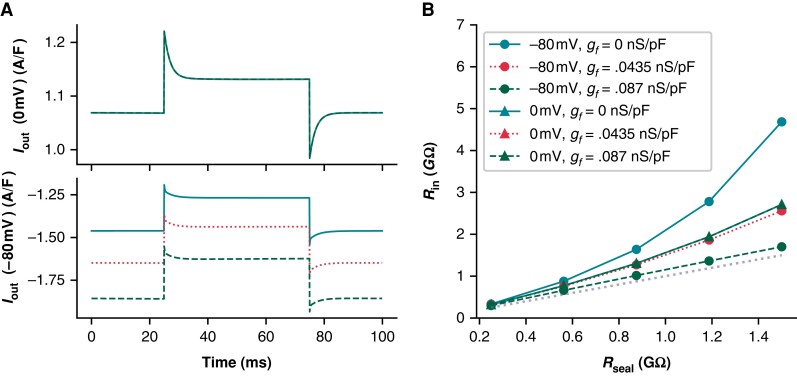
*R*_in_ predictions of *R*_seal_ are overestimated at the reversal potential for leak current. (*A*) The current response (*I*_out_) for Kernik + leak models with a 1 GΩ seal and *g_f_* of 0 (solid line), 0.0435 (dotted line), or 0.087 nS/pF (dashed line) to a 50 ms + 5 mV voltage clamp step from 0 mV (top) or −80 mV (bottom). (*B*) Effect of *R*_seal_ on *R*_in_ measures for models with *g_f_* set to 0 (solid), 0.0435 (dotted), or 0.087 nS/pF (dashed). *R*_in_ was calculated with Eq. (3). The +5 mV voltage steps were taken from either 0 or −80 mV. The *R*_seal_ = *R*_in_ line (dotted, without symbols) is provided as a reference for when *R*_in_ correctly predicts *R*_seal_. The 0 mV lines are overlapping, illustrating that *R*_in_ is not sensitive to *g_f_* at this voltage. The *g_f_* = 0.0875 nS/pF model at −80 mV provides the best estimate of *R*_seal_.

We recently showed that *I_f_* is present in at least some of the iPSC-CMs used in this study.^25^*I_f_* is also present in both the Kernik and Paci models, and we found the dynamics of the Kernik *I_f_* model to be quite similar to the *in vitro* data in this study (*Figure 5A* and *B*). *Figure 5A* shows an example cell’s response to an *I_f_*-activating hyperpolarizing step before and after treatment with quinine, at a concentration expected to lead to 32% *I_f_* block (these data are taken from a section of a larger protocol—see Clark *et al*.^25^*Figure 6A*). A change in total current of nearly 2 A/F is observed after holding at −120 mV for 1 s (*Figure 5A*). In Clark *et al*.,^25^ nine cells were treated with quinine, and the average change during the *I_f_*-activating segment was 1.34 A/F. We found that these nine cells could be sorted into three triplets based on the amount of quinine-induced *I*_out_ change during the *I_f_* segment: no/little sensitivity (Δ*I*_out_ of 0–0.2 A/F), moderate sensitivity (Δ *I*_out_ of 0.7–1.2 A/F), and large sensitivity (Δ *I*_out_ of >1.9 A/F). Simulations using the Kernik model with 32% block of *I_f_* show a change of 1 A/F (i.e. moderate change) in *I*_out_ (*Figure 5B*).

To illustrate the effect of *I_f_* on leak calculations, we compared simulations from Kernik + leak models with *R*_seal_ = 1 GΩ and with *g_f_* set to zero (i.e. not sensitive to quinine during hyperpolarizing step), the Kernik baseline value (*g_f_* = 0.0435 nS/pF, i.e. moderate sensitivity), or twice its baseline value (*g_f_* = 0.087 nS/pF, i.e. large sensitivity) (*Figure 5C*). We also reduced *g_K_*_1_ in these models to 10% of the baseline value to highlight the effects of *I_f_* on *R*_in_ measures independent of *I_K_*_1_. The calculated *R*_in_ values for these models at −80 mV are 2.03 GΩ for *g_f_* = 0 nS/pF (little change), 1.50 GΩ for *g_f_* = 0.0435 nS/pF (moderate change), and 1.16 GΩ for *g_f_* = 0.087 nS/pF (large change) (*Figure 5C*). These simulations show that, at −80 mV, *I_f_* contributes to *I*_out_ and affects measures of *I*_leak_.

Using these same models, we then calculated *R*_in_ values at multiple holding potentials between −90 and +30 mV to determine whether we could find a potential where *R*_in_ is close to *R*_seal_, thereby minimizing the prediction error (*Figure 5D*). The model predicts that 20 mV (*R*_in_ = 0.96 GΩ) minimizes the error in our approximation of *R*_seal_. This does not mean that *R*_in_ measurements at 20 mV will always produce the best estimate of *R*_seal_. Instead, it indicates the size of *I*_ion_ does not change much when taking a 5 mV step from this potential. There is, however, a considerable amount of total current present, making this *R*_seal_ prediction sensitive to variations in the predominant ionic currents at this potential. Moreover, *I*_leak_ will be small and therefore more difficult to measure as 10 mV is close to the leak reversal potential (0 mV). It is also worth noting that the complex voltage- and time-dependent behaviour of transmembrane currents make *R*_in_ measures sensitive to both the duration and size of the voltage step (e.g. see supplement to Clerx *et al*.^27^). In summary, it is difficult to find a holding potential where *R*_seal_ can be measured without contamination from any transmembrane currents (i.e. where *I*_leak_ = *I*_out_).

Taken together, these findings provide evidence to the claim that *R*_seal_ cannot be reliably measured in iPSC-CMs once access is gained.

Next, we compared the effect of *I_f_* on *R_m_* and investigated the error in assuming *R*_seal_ ≈ *R*_in_, at both a 0 mV (i.e. *I*_leak_ reversal) and −80 mV holding potential. At 0 mV, the Kernik + leak model is not sensitive to changes in *g_f_*, as *I_f_* is largely non-conductive (*Figure 6A*). However, due to an increased relative contribution of inward currents at 0 mV, the Kernik + leak model predicts a *R*_in_ with a large overestimation of *R*_seal_ (*Figure 6B*). This error increases as the true value of *R*_seal_ increases. *Figure 6B* also illustrates the sensitivity of the model to variations in *g_f_* at −80 mV, with *R*_seal_ estimation errors decreasing as *g_f_* increases; these errors also increase as *R*_seal_ increases. The improved prediction accuracy of the 0.087 nS/pF model at −80 mV is a coincidental side effect of doubling *g_f_*: with a different distribution of ion current densities or a larger baseline *g_f_* value, the same doubling could just as easily worsen *R*_seal_ predictions. For example, the *R*_in_ of an iPSC-CM with a large *I_K_*_1_ current may slightly underestimate *R*_seal_ at −80 mV—doubling *g_f_* in this case would result in a greater underestimation, increasing the error of the estimate.

### *C_m_* and *R*_in_(0 mV) correlate with minimum potential

**Figure 7:**
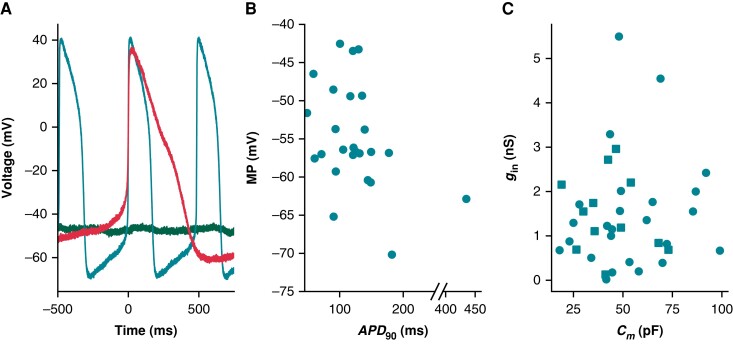
Cells appeared phenotypically heterogeneous, with uncorrelated variation in *g*_in_ and *C*_m_. (*A*) Current clamp recordings from three cells show phenotypic heterogeneity: non-spontaneous (green), spontaneous AP with short APD (teal), and spontaneous AP with long APD (red). (*B*) MP and *APD*_90_ for spontaneously beating cells (*n* = 25). Note the broken *x*-axis that allows us to display an outlying data point. (*C*) The relationship between *C*_m_ and *g*_in_ for all cells (*n* = 37). Non-spontaneous cell data points are denoted with squares, whilst spontaneous are circles. *APD*_90_, action potential duration at 90% repolarization; MP, minimum potential.

The iPSC-CMs used in this study displayed a heterogeneous phenotype (*Figure 7*), producing both spontaneously firing (*n* = 25) and non-firing (*n* = 12) current clamp recordings. *Figure 7A* shows three cells with very different baseline current clamp recordings: non-firing and depolarized (green), spontaneously firing with a short AP (teal), and spontaneously firing with a long AP (red). Non-firing cells (MP = −42 ± 8 mV) and cells with spontaneously firing APs were depolarized (MP = −54 ± 7 mV)—the spontaneously firing cells also had a shorter AP duration (*APD*_90_ = 128 ± 71 ms) (*Figure 7B*) relative to adult cardiomyocytes^28^ and iPSC-CM models.^13,14^

We used linear regression analyses to determine if there is a correlation between *g*_in_/*C_m_* and AP biomarkers. Here, we use *g*_in_ (instead of *R*_in_), as it reduces the spread of this variable and positively correlates with *I*_leak_ providing a more interpretable comparison with AP morphology. The values of each cell’s *g*_in_ and *C_m_* are shown in *Figure 7C*. *I*_leak_’s effect on AP morphology is expected to scale directly with *g*_in_ and inversely with *C_m_*. This is because *g*_in_, even if a poor estimate, is expected to correlate with *g*_seal_ (*Figure 6B*)

**Figure 8:**
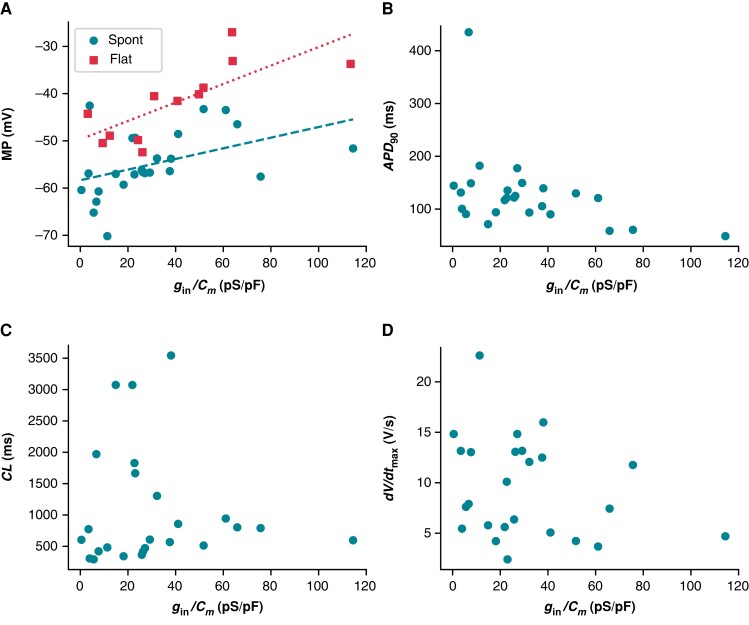
Relationship between *g*_in_/*C*_m_ and AP biomarkers. (*A*) *g*_in_/*C*_m_ plotted against MP. Spontaneously firing cells are denoted as teal circles and non-firing cells as red squares. Linear regression fits to data from spontaneous (teal dashed, *R* = 0.47, *P* < 0.05), and non-firing (red dotted, *R* = 0.76, *P* < 0.05) cells are overlaid on the plot. No statistically significant relationship was found between *g*_in_/*C*_m_ and *APD*_90_ (*B*), CL (*C*), or *dV*/*dt*_max_ (*D*). *APD*_90_, action potential duration at 90% repolarization; CL, cycle length; *dV*/*dt*_max_, maximum upstroke velocity; MP, minimum potential.

A given *g*_leak_ will cause a smaller contribution in larger cells (i.e. cells with larger *C_m_*), because the ionic currents are expected to scale with the size of the cell. For this reason, four AP biomarkers (MP, *APD*_90_, CL, and *dV*/*dt*_max_) were compared with *g*_in_/*C_m_* (*Figure 8*). The MPs of spontaneously firing (*R* = 0.44, *P* < 0.05) and non-firing (*R* = 0.76, *P* < 0.05) cells are positively correlated with *g*_in_/*C_m_* (*Figure 8A*). This finding is in agreement with our *in silico* studies showing that increasing *g*_seal_, thereby increasing *g*_in_, will depolarize the cell (*Figure 2*). The other three biomarkers failed at least one of the assumptions required when conducting a linear regression analysis (see *Supplementary Methods*). There are no obvious trends when comparing *g*_in_/*C_m_* with CL or *dV*/*dt*_max_. The *APD*_90_ plot, however, indicates there may be some AP shortening as *g*_in_/*C_m_* increases. Due to under-sampling and a lack of linearity, we cannot make any claims of significance between these two measures. Leak simulations with the models, though correlated, did not predict a linear relationship between *g*_seal_ and these biomarkers (*Figure 2C* and *D*). However, the MP vs. *g*_in_/*C_m_* relationship passes all tests of linear regression assumptions and trends in the same direction as the Kernik and Paci simulations in *Figure 2*.

### Fitting background currents in human-induced pluripotent stem cell-derived cardiomyocyte models can absorb and imitate *I*_leak_

**Figure 9:**
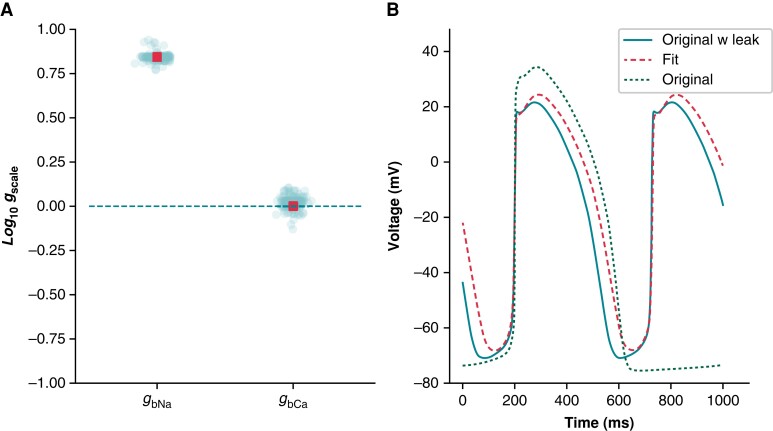
A simulated example of how leak can be absorbed into background currents: Kernik baseline model fit to Kernik + leak model. The *I*_bNa_ and *I*_bCa_ conductances (*g*_bNa_ and *g*_bCa_) of the baseline Kernik model were fit to a Kernik + leak model (i.e. original + leak) with *R*_seal_ set to 5 GΩ using a genetic algorithm. (*A*) The conductances for all individuals (teal circles) and the best fit individual (red square) from the last generation. (*B*) Traces from the original baseline Kernik + leak model with a 5 GΩ seal (teal solid), the best fit model from the last generation (red dashed), and the original baseline Kernik model (green dotted).

We used optimization to study the potential of linear background currents (e.g. sodium and calcium) to imitate leak effects (see *Supplementary Methods*). We fit the baseline Kernik model to a Kernik + leak model with *R*_seal_ = 5 GΩ (*Figure 9*), allowing only the background sodium (*g*_bNa_) and background calcium (*g*_bCa_) conductances to vary. These currents were selected because they were incorporated into the Kernik model without independent iPSC-CM experimentation or validation. The best-fit model had an increased *g*_bNa_ (×7.0), whilst *g*_bCa_ (×1.0) did not change much relative to the baseline model (*Figure 9A*). Whilst not a perfect match, the best-fit trace reproduced qualitative features of the baseline + leak trace, showing a depolarized MP and a smaller amplitude (*Figure 9B*). This indicates that increased *I*_bNa_ can affect the AP in a fashion similar to *I*_leak_ such that mathematical iPSC-CM models may absorb *I*_leak_ effects by erroneously increasing background currents.

## Discussion

Leak current is a common and unavoidable experimental artefact that affects patch clamp recordings. In this study, using both model predictions and experimental data, we show that leak current: (i) affects iPSC-CM AP morphology, (ii) can vary during experiments, (iii) cannot be accurately estimated after access is gained to an iPSC-CM, and (iv) may be absorbed by linear equations for background currents when iPSC-CM models are fit to experimental AP data. During iPSC-CM current clamp studies, leak consideration often starts with a pre-rupture seal measurement (with a 1 GΩ threshold) and is ignored if the seal appears to remain stable throughout the study. Here, we argue leak effects should be quantitatively scrutinized during the acquisition, analysis, and fitting of experimental data. Furthermore, we believe cell-to-cell variation in seal resistance contributes to observed iPSC-CM AP heterogeneity—often attributed nearly entirely to variations in ionic current densities.

### Leak affects action potential morphology

Simulations in chick embryonic cardiomyocytes, which are smaller than adult human cells (with model *C_m_* = 25.5 pF), have previously shown that leak current substantially depolarizes the MP and shortens the CL, even with *R*_seal_ values of 5 GΩ.^29^ More recently, it was shown that *in vitro* iPSC-CMs were significantly depolarized during single-cell experiments, but not when cells were clustered.^11,12^ These results indicate that isolated iPSC-CMs are likely affected by leak current. Our *in vitro* and *in silico* findings support this conclusion and strengthen the argument that iPSC-CM AP morphology is strongly affected by leak current.

Our *in silico* work indicates that *I*_leak_ has a smaller effect on recordings of adult cardiomyocyte AP morphology when compared with iPSC-CMs (*Figure 3B*). This effect is strongly modulated by *C_m_*, indicating the larger size of adult cardiomyocytes has a moderating effect on *I*_leak_-induced AP changes. When the *I*_leak_ artefact in this adult model is normalized by the average iPSC-CM capacitance (50 pF, *Figure 3A*), *I*_leak_ substantially alters the AP shape at *R*_seal_ values above 1 GΩ. But the effects are much less than in the iPSC-CM model (*Figure 2*)—this indicates the ionic current expression profile of adult cardiomyocytes (e.g. greater *I_K_*_1_ and lower *I_f_* density), in addition to cell size, and moderates the effects of *I*_leak_ on adult AP recordings. Thus, differentiation strategies that aim to mature the iPSC-CM phenotype (both in size and ionic current expression) will likely produce cells that are affected less by *I*_leak_ artefact.

Human-induced pluripotent stem cell-derived cardiomyocytes have long been defined by their immature and heterogeneous electrophysiological phenotype.^10,30^ Such features are due, at least in part, to the types of ion channels expressed and cell-to-cell variations in ionic current conductances.^10,30^ In this study, differences in the *I_f_* responses to nine quinine-treated cells are an example of how iPSC-CM ionic currents can vary from one cell to the next. Heterogeneity in AP morphology and ionic current expression is also seen in primary adult cardiomyocytes.^31–33^

In this study, we show that *I*_leak_ also contributes to this immature and heterogeneous AP phenotype during single-cell patch clamp experiments. The relative importance of *I*_leak_’s influence on AP shape varies amongst cells and depends on several factors, including *R*_seal_, *C_m_*, and the ionic current expression profile. Simulations indicate that the AP shape can be substantially altered (relative to non-patched cells), even when *R*_seal_ is equal to 10 GΩ, an unrealistically high acceptance criterion for iPSC-CM patch clamp studies. These factors, along with the potential for *R*_seal_ to change during an experiment, can confound drug and genetic mutation studies. For example, the irregular and depolarized phenotype (caused at least in part by *I*_leak_) of iPSC-CMs in our recent cardiotoxicity study^25^ made it impossible to measure consistent cell-specific changes in spontaneous AP morphology from pre- to post-drug application.

The AP-altering effects of *I*_leak_ can be effectively eliminated by patching cells whilst in engineered heart tissue or monolayer. The electrical coupling of cells in these conditions results in an enormous effective capacitance, rendering *I*_leak_ an infinitesimal contributor to total current. Whilst this eliminates the *I*_leak_ artefact, it also comes at a cost—this approach does not allow for the direct measure of APs in individual cells, limiting the ability to study iPSC-CM heterogeneity. In addition, it is not possible to acquire voltage clamp data from cells in these conditions—as such, one could not acquire both AP and descriptive data about individual currents, as we recently have done in isolated cells.^25^

### Predicting *R*_seal_ during experiments

*R*_seal_ can be well approximated prior to gaining access to a cell, but after perforation (or rupture), the presence of membrane currents makes it impossible to obtain an accurate measurement (*Figure 5*). Our *in silico* work shows that, even when currents such as *I_f_* and *I_K_*_1_ are reduced to <10% of their baseline values, *R*_in_ (measured at −80 mV) is still a poor approximation of *R*_seal_ (*Figure 6*, solid black line).

To address these difficulties, we believe it may be feasible to use the pre-rupture *R*_seal_ and post-rupture *R*_in_ measures to calculate estimates of *R*_seal_ during an experiment. This approach would require an accurate measure of *R*_in_ just after access is gained. Using *R*_seal_ and the initial *R*_in_, it is possible to calculate *R_m_* (*Figure 1*). An estimate of *R*_seal_ could then be made at any time during the experiment, assuming the calculated *R_m_* stays constant, by re-measuring *R*_in_ and using Eq. (4). This approach relies on two major assumptions: (i) the perforation/rupture step does not affect the seal, and (ii) a protocol or procedure exists that can be used prior to each measurement of *R*_in_ to ensure that the contribution of *R_m_* is consistent. We cannot say for certain that these assumptions will always be valid. However, we believe that recording frequent *R*_in_ measurements, estimating *R*_seal_, and scrutinizing changes are important steps for the correct interpretation of iPSC-CM current clamp data.

### Correcting for *R*_seal_ during experiments

We believe these *R*_seal_ estimates should be used in a dynamic clamp leak compensation setup to address the limitations caused by a depolarized and variable MP. The approach works by injecting simulated currents into a cell in a real-time continuous loop during current clamp experiments.^34^*I_K_*_1_ dynamic clamp has been used on iPSC-CMs to attain quiescence at a MP below −70 mV so the cells can be paced at a desired frequency.^25,35–37^ A dynamically clamped leak compensation current has been implemented and used in manual patch clamp studies with neonatal mouse cardiomyocytes,^22^ demonstrating the potential of using such an approach with small cardiomyocytes. The effects of leak and the ability of leak compensation to recover adult cardiomyocyte behaviour have also been demonstrated in an *in silico* study.^23^ Together, these investigations demonstrate the potential of dynamic clamp as an experimental tool to simultaneously address shortcomings of the cells (i.e. *I_K_*_1_ density) and experimental setup (i.e. *I*_leak_). This technique has the potential to improve the descriptive ability of iPSC-CMs when used in biophysical and drug investigations.

Inaccuracies in these estimates, however, will remain, resulting in the potential to under- or over-compensate. Over-compensation will hyperpolarize the MP and prolong Phases 1 and 2 of the AP, so we believe under-compensation is preferable. We suggest injecting a fraction of the full compensatory current to mitigate the risk of underestimating *R*_seal_. The Nanion Dynamite^8^ sets the leak per cent compensation to 70%, which seems reasonable.^38^

### Models of background currents can incorporate leak artefacts

The Kernik and Paci iPSC-CM models took ion-specific background currents from the ten Tusscher *et al*.^39^ model. These currents can trace their roots to the seminal work of Luo *et al*.,^40^ where they were included to help maintain physiologically realistic intra-cellular concentrations.

Direct measurements of *I*_bCa_ and *I*_bNa_ in iPSC-CMs have not been reported. The Kernik and Paci iPSC-CM models both adopted the ventricular^39^ formulation for *I*_bCa_ and *I*_bNa_ and then set the conductances of these currents by comparing model predictions of the AP with *in vitro* measurements in iPSC-CMs. We posit that *I*_bNa_ is overestimated and compensates for the explicit consideration of leak current artefacts, a source of discrepancy between these models and reality. We expect consideration of leak when constructing iPSC-CM models to reduce background sodium current and result in a more realistic model of intact iPSC-CMs.

### Modelling experimental artefacts

Whilst the effects of experimental artefacts in single-cell studies are well-established, consideration of them whilst building ion channel and AP models has been limited.^41^*In silico* studies investigating series resistance effects on voltage clamp recordings have been done in fast-activating currents, such as *I*_Na_ and *I*_to_,^42,43^ but to our knowledge, artefact equations have not been included in the calibration process for widely used models of these currents—although the *I*_Na_ model by Ebihara *et al*.^42^ was incorporated directly into the widely copied *I*_Na_ model by Luo *et al*.^40^ Recently, Lei *et al*.^44^ demonstrated that coupling experimental artefact equations with an *I*_Kr_ mechanistic model improved predictions. These studies show that including experimental artefact equations in model fitting can improve the descriptive ability of the resulting electrophysiological models. As such, we believe experimental artefacts should be explicitly considered at the modelling phase and not ignored simply because a pre-determined minimum threshold is reached (e.g. 1 GΩ). Based on our findings, we believe cardiomyocyte models and especially iPSC-CM models should explicitly include leak currents when fitting to experimental current clamp data.

### Recommendations

Our results provide important insights and recommendations for experimentalists and modellers alike:

1. *Experimental*: *R*_seal_ should be recorded before gaining access to a cell and *R*_in_ measured frequently during an experiment. It is important to measure *R*_in_ from a voltage that provides a consistent measure of *R_m_*, such that any changes in *R*_in_ can be attributed to changes in *R*_seal_.
2. *Experimental*: Dynamic injection of a leak compensation current can help a cell recover its native AP, including the MP. Because *R*_seal_ is difficult to measure during experiments and to avoid over-compensation, we advise under-compensation (e.g. 70%). Additionally, *R*_seal_ and *R*_in_ measures should be reported.
3. *Modelling*: Explicit inclusion of *I*_leak_ will improve the descriptive ability of iPSC-CM models. Whilst this may not always improve fits to AP data, it will take into account an important current affecting iPSC-CM recordings.

### Limitations and future directions

This study has several limitations that should be considered during future investigations that may be affected by *I*_leak_. First and foremost, when gathering these data for a previous study, we did not follow our new recommendation of recording the exact value of *R*_seal_ before gaining access and then measuring *R*_in_ just after perforation. Going forward, we hope to use these two values to predict *R*_seal_ at multiple time points during an experiment, as outlined in Section 3.2. Second, we only conducted these experiments in one cell line. Whilst our results appear similar to data from other labs,^11^ it would be useful to conduct this study on multiple cell lines in the same lab. Third, we did not attempt dynamic injection of a leak compensation current—in future work, we would like to investigate this as an approach to reducing cell-to-cell heterogeneity. Finally, the iPSC-CM models have innumerable differences from the cells used in this study, which is evident when comparing AP morphologies of *in vitro* cells (*Figure 7A*) to *in silico* models (*Figure 2*). However, the agreement that we did see between simulations and our *in vitro* data demonstrates the potential of improving the descriptive ability of iPSC-CM models by including a leak current.

### Conclusion

In this study, we demonstrate that leak current affects iPSC-CM AP morphology, even at seal resistances above 1 GΩ, and contributes to the heterogeneity that characterizes these cells. Using both *in vitro* and *in silico* data, we showed the challenges of estimating *R*_seal_ after gaining access to a cell and that *R*_seal_ is subject to change during the course of an experiment. We also posit that background sodium current in iPSC-CM models may be responsible for masking leak effects in *in vitro* data. Based on these results, we make recommendations that should be considered by anyone who collects, analyses, or fits iPSC-CM AP data.

## Supplementary Material

euad243_Supplementary_Data

## Data Availability

All data, code, and models can be accessed from GitHub (https://github.com/Christini-Lab/iPSC-leak-artifact).

## Translational perspective

Human iPSC-CMs have emerged as a promising translational tool to study human cardiac physiology outside of the clinic. They have been particularly useful to investigate cell-level pro-arrhythmic substrates, including genetic mutations and ion channel-blocking drugs, and play a critical role as a model for validating drug effects on human whole-cell electrophysiology in the Comprehensive *in vitro* Proarrhythmia Assay (CiPA) initiative. However, the depth of insights from iPSC-CM data is often limited by inter- and intra-lab heterogeneity caused, at least in part, by patch clamp experimental artefact. In this manuscript, we show how the seal–leak current is an often-overlooked artefact that confounds studies with iPSC-CMs. Ultimately, the findings and recommendations within this manuscript will improve the use of iPSC-CMs as an *in vitro* model to study cardiac electrophysiological diseases and patient-specific treatment strategies.
