# Supplementary material for: Leak current, even with gigaohm seals, can cause misinterpretation of stem cell-derived cardiomyocyte action potential recordings
Source: Europace. 2023 Aug 8;25(9):euad243. doi: 10.1093/europace/euad243 (PMC10445319; doi:10.1093/europace/euad243)
Supplement: euad243_Supplementary_Data [file euad243_supplementary_data.pdf]

Supplementary Material

Leak current, even with gigaohm seals, can cause misinterpretation of stem cell-derived cardiomyocyte action potential recordings

Alexander P. Clark, Michael Clerx, Siyu Wei, Chon Lok Lei,
Teun P. de Boer, Gary R. Mirams, David J. Christini, Trine Krogh-Madsen

---

1 Supplementary methods

1.1 Fixing intracellular concentrations

I_{leak} can destabilise intracellular concentrations and cause a slow and continuous decrease in intracellular potassium concentration, $[K^+]_i$. To address this, we fixed the Kernik and ToR-ORd $[K^+]_i$ to their respective steady state values. This was not required for the Paci model, which does not allow $[K^+]_i$ to change. We also fixed the $[Na^+]_i$ to baseline steady state values (taken after 1000 s of spontaneous or paced current clamp simulation). We did not fix the intracellular calcium concentration, because it is less affected by the pipette solution during perforated patch-clamp experiments with amphotericin B as only monovalent ions can diffuse through the pores.

1.2 Optimization

A genetic algorithm (GA) was used to fit the Kernik model to the Kernik+leak model (with R_{seal}=5 GΩ) by minimising a point-by-point squared difference objective function:

$$E_{in}(\theta) = \sum_{t=0}^{1000} \left(V_{target}(t) - V_{individual}(t,\theta)\right)^2, \tag{1}$$

where θ is a vector containing the varied conductance parameters, $V_{target}(t)$ is the target membrane potential at time t, and $V_{individual}(t,\theta)$ is the current individual's membrane potential at time t as a function of θ.

The GA had a population size of 150 individuals and was run for 20 generations. The initial population parameter values were selected from a log uniform distribution between 0.1 and 10 times their baseline values. Individuals in a new generation were created by mating two individuals from the previous generation — two selected parent individuals from the previous generation had a 90% chance of mating. If they did not mate, they would continue

to the next generation without swapping parameters. If they mated, there was a 20% chance of swapping each of their parameter values. As such, each time two individuals mated, they would produce two child individuals consisting of the parent parameter values. Each individual in a new generation had a 90% chance of being mutated. If an individual was mutated, there was a 20% chance each parameter would be changed. To mutate a parameter, a new value was selected from a normal distribution centred around the current value, with a standard deviation equal to 10% of the current value.

The Kernik+leak target and each individual were run for 100 s before comparison. The third-to-last AP was identified from each, and traces were aligned by the dV/dt_{max} of these APs. Traces were compared from 200 ms before the dV/dt_{max} to 800 ms after it. The code for this GA can be found on the project GitHub page.

1.3 Linear regression

A linear least-squares regression was used to compare g_{in}/C_m to AP biomarkers. MP was the only biomarker that did not violate any linear regression assumptions when compared to these independent variables. Tests of these assumptions can be found on the project GitHub repository.

1.4 Software and simulations

Simulations were performed in Myokit v1.33.7[1]. The genetic algorithm was developed in Python and made use of the DEAP library v1.2.2[2]. Additional analysis was done in Python using NumPy v1.21.6 and SciPy v1.7.3[3].

1.5 iPSC-CM cell culture

Frozen vials of iPSC-CMs were obtained from Joseph C. Wu, MD, PhD at the Stanford Cardiovascular Institute Biobank. The iPSC-CM line was derived from an African American female donor and the differentiation was approved by the Stanford University Human Subjects Research Institutional Review Board. Cells were prepared for electrophysiological experiments following the steps described in[4]. Briefly, cells were thawed and cultured as a monolayer in one well of a 6-well plate precoated with 1% Matrigel. Cells were cultured with RPMI media (Fisher/Corning 10-040-CM) containing 5% FBS and 2% B27 and kept in an incubator at 37 °C, 5% CO_2, and 85% humidity. After 48 hours, cells were lifted with 1 mL Accutase, diluted to 100,000 cells/mL, and replated on 124 sterile 8 mm coverslips precoated with 1% Matrigel. Cells were cultured with RPMI media that was swapped every 48 hours. Cells were patched between days 5 and 15 after thaw.

1.6 Electrophysiological setup

Perforated patch-clamp experiments were conducted following the protocol described in[4]. Borosilicate glass pipettes were pulled to a resistance of 2-4 MΩ using a flaming/brown micropipette puller (Model P-1000; Sutter Instrument, Novato, CA). Pipette tips were first dipped into intracellular solution containing 10 mM NaCl, 130 mM KCl, 1 mM $MgCl_2$, 10 mM $CaCl_2$, 5.5 mM dextrose, 10 mM HEPES. Pipettes were then backfilled with intracellular solution with 0.44 mM amphotericin B, a perforating agent. Amphotericin B allows only monovalent

ions to pass through the cell membrane, so a high intrapipette calcium concentration was included to induce cell death in the case of an unintended rupture. Coverslips containing iPSC-CMs were placed in the bath and constantly perfused with an extracellular solution at 35-37℃ containing 137 mM NaCl, 5.4 mM KCl, 1 mM $MgSO_4$, 2 mM $CaCl_2$, 10 mM dextrose, and 10 mM HEPES.

Patch-clamp measurements were made at a 10 kHz sampling frequency by an amplifier with the low-pass filter set to 5 kHz (Model 2400; A-M Systems, Sequim, WA), and was controlled by the Real Time eXperiment Interface (RTXI; http://rtxi.org). After immersing a pipette into the extracellular solution, voltage was set to zero — any remaining offset in the recordings is assumed to be equal to the liquid junction potential of −2.8 mV. After contact was made with a cell and a seal of > 300 MΩ was formed, the perforating agent slowly decreased the access resistance to the cell (usually 10–15 minutes). This low R_{seal} acceptance criterion was selected because we wanted to explore seal-leak effects above and below 1 GΩ. A series resistance of 9–50 MΩ was maintained for all experiments. After gaining access, R_m at 0 mV was measured before and after acquiring AP data.
